# Post-implantation analysis of genomic variations in the progeny from developing fetus to birth

**DOI:** 10.1186/s40246-024-00634-4

**Published:** 2024-07-15

**Authors:** Yingming Zheng, Chuanping Lin, Wen-Jing Wang, Liya Wang, Yeqing Qian, Luna Mao, Baohua Li, Lijun Lou, Yuchan Mao, Na Li, Jiayong Zheng, Nan Jiang, Chaying He, Qijing Wang, Qing Zhou, Fang Chen, Fan Jin

**Affiliations:** 1 Department of Reproductive Endocrinology, Key Laboratory of Reproductive Genetics of National Ministry of Education, Women’s Reproductive Health Laboratory of Zhejiang Province, Women’s Hospital, School of Medicine, Zhejiang University, 1 Xueshi Road, Hangzhou, Zhejiang 310006 China; 2 Reproductive Medical Center, the Second Affiliated Hospital of Wenzhou Medical College and Yuying Children’s hospital, Wenzhou, Zhejiang 325027 China; 3 BGI Research, Shenzhen, Guangdong 518083 China; 4 Affiliated Dongyang Hospital of Wenzhou Medical University, Dongyang, Zhejiang 322100 China; 5 Reproductive Medical Center, the First Affiliated Hospital, School of Medicine, Zhejiang University, Hangzhou, Zhejiang 310003 China; 6 Hangzhou Women’s Hospital (Hangzhou Maternity and Child Health Care Hospital), Hangzhou, Zhejiang 310008 China

**Keywords:** Whole-genome sequencing (WGS), SNV, *De novo* indels, Newborns

## Abstract

The analysis of genomic variations in offspring after implantation has been infrequently studied. In this study, we aim to investigate the extent of *de novo* mutations in humans from developing fetus to birth. Using high-depth whole-genome sequencing, 443 parent-offspring trios were studied to compare the results of *de novo* mutations (DNMs) between different groups. The focus was on fetuses and newborns, with DNA samples obtained from the families’ blood and the aspirated embryonic tissues subjected to deep sequencing. It was observed that the average number of total DNMs in the newborns group was 56.26 (54.17–58.35), which appeared to be lower than that the multifetal reduction group, which was 76.05 (69.70–82.40) (F = 2.42, *P* = 0.12). However, after adjusting for parental age and maternal pre-pregnancy body mass index (BMI), significant differences were found between the two groups. The analysis was further divided into single nucleotide variants (SNVs) and insertion/deletion of a small number of bases (indels), and it was discovered that the average number of *de novo* SNVs associated with the multifetal reduction group and the newborn group was 49.89 (45.59–54.20) and 51.09 (49.22–52.96), respectively. No significant differences were noted between the groups (F = 1.01, *P* = 0.32). However, a significant difference was observed for *de novo* indels, with a higher average number found in the multifetal reduction group compared to the newborn group (F = 194.17, *P* < 0.001). The average number of *de novo* indels among the multifetal reduction group and the newborn group was 26.26 (23.27–29.05) and 5.17 (4.82–5.52), respectively. To conclude, it has been observed that the quantity of *de novo* indels in the newborns experiences a significant decrease when compared to that in the aspirated embryonic tissues (7–9 weeks). This phenomenon is evident across all genomic regions, highlighting the adverse effects of *de novo* indels on the fetus and emphasizing the significance of embryonic implantation and intrauterine growth in human genetic selection mechanisms.

## Introduction

The mechanisms of genetic selection are of utmost importance in the progression of human reproduction, encompassing the maturation of gametes, formation of fertilized eggs, emergence of cleavage embryos, development of blastocysts, implantation of embryos, and formation of pregnancy embryos, ultimately culminating in the birth of offspring. The precise expression of genetic material serves as the fundamental basis for the normal development of progeny [1]. Genomic variations, which are directed by the reference of the genome sequence, encompass modifications to the structural base pair composition or sequence arrangement of genes. These includes copy number variations (CNVs), single nucleotide variants (SNVs), and insertion/deletion of a small number of bases (indels) [2]. The occurrence of *de novo* SNVs and indels has been reported at an average rate of approximately 70 *de novo* mutations (DNMs) per individual [3]. These variations represent significant targets for the operation of genetic selection mechanisms.

Certain genomic variations that are specific to certain regions or types can result in various complications during pregnancy, including but not limited to oocyte maturation impairment, fertilization anomalies, diminished embryonic developmental potential, biochemical pregnancies, and miscarriages, which ultimately lead to pregnancy termination [4]. It is widely accepted that embryonic chromosomal abnormalities are the primary cause of early pregnancy loss, accounting for more than 50% of early miscarriages. These abnormalities can include anomalies in chromosome numbers and structure, such as microdeletions and duplications of chromosomal fragments [5, 6]. By means of genetic selection, the majority of offspring with severe illnesses and developmental abnormalities are effectively prevented from being born, thereby significantly enhancing the genetic stability of the human population. Gaining a deeper understanding of how the quantity of each type of genomic variation evolves during offspring development can facilitate our comprehension of this mechanism and enable more targeted research on the genetic safety of offspring in clinical practice.

The genetic selection mechanism, in fact, plays a crucial role in all stages of embryonic development. With the advent of assisted reproductive technology (ART), individuals now have the opportunity to conduct in vitro research on gametes and embryos at all stages of development. Furthermore, the introduction of whole genome sequence (WGS) technology has spurred investigations of DNMs in humans at the whole genome level. Based on the available literature, the majority of studies have focused on the genomic variation of oocyte, cleavage embryos, and blastocysts. Previous research, which included copy number variation sequencing (CNV-seq) data [7–9], has demonstrated that high frequency aneuploidy and large fragment (> 2 Mb) of pathogenic CNVs were present in oocytes, cleavage embryos and blastocysts, but were largely absent in reduced fetal tissue during early implantation of embryos. However, there is limited research that has delved deeply into chromosomal variation from fetus to birth after implantation, and even less is known about the timing and frequency of these variants. A recent study of limited scope has provided evidence indicating that there were no significant differences observed in the number of DNMs per child for various methods of conception [10]. As such, the present study aims to estimate the differences in *de novo* variation between early gestational fetal tissue and newborns when subsequent to implantation. DNMs which are known to cause most human genetic diseases, some adverse perinatal outcomes, and congenital and developmental diseases of the offspring, were selected as the target of this study [11]. WGS was conducted on parent-offspring trios to identify DNMs.

## Materials and methods

### Ethical approval and study subjects

The present study was conducted under the auspices of a license obtained from the Human Genetic Resource in China ([2021] CJ0522). The Institutional Review Board of the School of Medicine, Zhejiang University, China, granted ethical approval for this project (approval number: 20,180,127), and written informed consent was obtained from all participants. The study population was divided into two groups: the multifetal reduction group and the newborn group.

#### The multifetal reduction group

Between December 2018 and July 2021, a total of 57 multifetal reduction tissues were collected. The study invited 46 couples who had undergone in in-vitro fertilization (IVF) and 11 couples who had undergone intracytoplasmic sperm injection (ICSI) to donate blood samples for DNA extraction to aid in ongoing research. The multiple pregnancies in question were a result of IVF and ICSI, and all fetuses were either triplets or part of a gemellary pregnancy. Multifetal reduction was performed at 7–9 weeks after the fetal heartbeat was observed, reducing the number of fetuses from triplets to twins or from twins to a singleton. The most accessible gestational sac was selected and aligned with the puncture guideline on the screen. Or choose to the fetus with a relatively small gestational sac, if the surgical path allows. After introducing the needle into the fetal echoes, suction was applied repeatedly using a 50-mL syringe until all fetal parts were aspirated. The analysis was conducted on a total fetus that did not contain chorionic villi from the volunteered reduction.

#### The newborn group

Between December 2018 and October 2020, a total of 306 families who had undergone ART were recruited for a study. This group consisted of 189 families who had undergone IVF and 117 families who had undergone ICSI, resulting in a total of 386 infants (234 IVF and 152 ICSI). The couples were invited to donate blood samples for DNA extraction to assist in ongoing research, and parental consent was obtained for the collection of umbilical cord blood from the offspring at the time of delivery. General paediatric examinations were conducted at birth to identify any obvious somatic abnormalities in the children.

The criteria for exclusion from participation in this study included the inability of any subject to undergo blood or tissue sampling, the presence of known chromosomal abnormalities in either parent, the use of IVF/ICSI following the donation of oocytes or semen, and a history of chemotherapy or radiation therapy for malignancy in either parent. The baseline characteristics of each individual were meticulously collected.

### Sequencing and variant calling

For each household, we extracted genomic DNA (gDNA) from both the father and mother, as well as from the multifetal reduction tissues or neonate umbilical cord blood following live birth (triple DNA sample set). WGS libraries were prepared using a Universal DNA Library Prep Set in accordance with the manufacturer’s protocol (MGI; Cat: 1,000,017,571). The gDNA was fragmented, ligated with an adaptor after end repair and A-tailing, amplified, and sequenced at the China National GeneBank. A minimum of 500 million 100 bp paired-end reads were obtained from each sample on the DNBSEQ-T1 platform.

After implementing quality control measures and filtering out low-quality reads, short variants of SNVs and indels of each sample were identified by the Sentieon pipeline [12] based on the human reference genome (GRCh37). Subsequently, we utilized the module of variants quality score recalibration (VQSR) within the Genome Analysis Toolkit (GATK v3.4.46) as described by McKenna, A., et al. (2010) [13]. This enabled us to acquire high-confidence variants for all autosomes and X chromosomes (tranche 0.99). For each trio, individual genome variant call format files (gVCF) were jointly genotyped using Genotype GVCFs, and variants that passed the filter of VQSR were obtained for further analysis.

### *De novo* mutation identification

We called DNMs using the DeNovoGear [14] TrioDeNovo [15] and GATK Genotype Refinement workflows [13]. The results that were found to be consistent by three pipelines were designated as DNM, provided they satisfied the following criteria: (1) passing the VQSR filter in the offspring; (2) genotype quality (GQ) greater than 90 in the offspring; (3) GQ greater than 30 in both parents; (4) read depth (DP) between 20 and 150 in the offspring; (5) DP greater than 20 in both parents; (6) alternative allele fraction greater than 0.2; and (7) located on the autosomes. To assess the accuracy of our identification method, we selected all DNMs from 5 trios and subjected them to Sanger sequencing (BGI TECH SOLUTIONS (BEIJING LIUHE) CO., LIMITED) to validate these variants. We extracted sequences in the vicinity of the variant sites and designed PCR primers for all these sites using the Primer Design Tool from NCBI.

### *De novo* mutation interpretation

The DNMs were systematically classified as pathogenic, likely pathogenic, benign, likely benign and uncertain significance, employing InterVar based on a multitude of factors including but not limited to allele frequencies across populations, evolutionary conservation metrics, and predictive functional annotations [16].

### Statistical analysis

The data have been presented as the mean value along with a 95% confidence interval [CI]. The Student’s t-test has been employed to analyze the data, which conforms to a Gaussian distribution. Furthermore, Chi-square tests have been utilized to compare count data across different groups. Additionally, a covariates test has been conducted to eliminate any potential impact of clinical background information. All statistical analyses were conducted using IBM SPSS 26.0. A significance level of *P* < 0.05 was deemed statistically significant.

## Result

### Clinical data

**Fig. 1:**
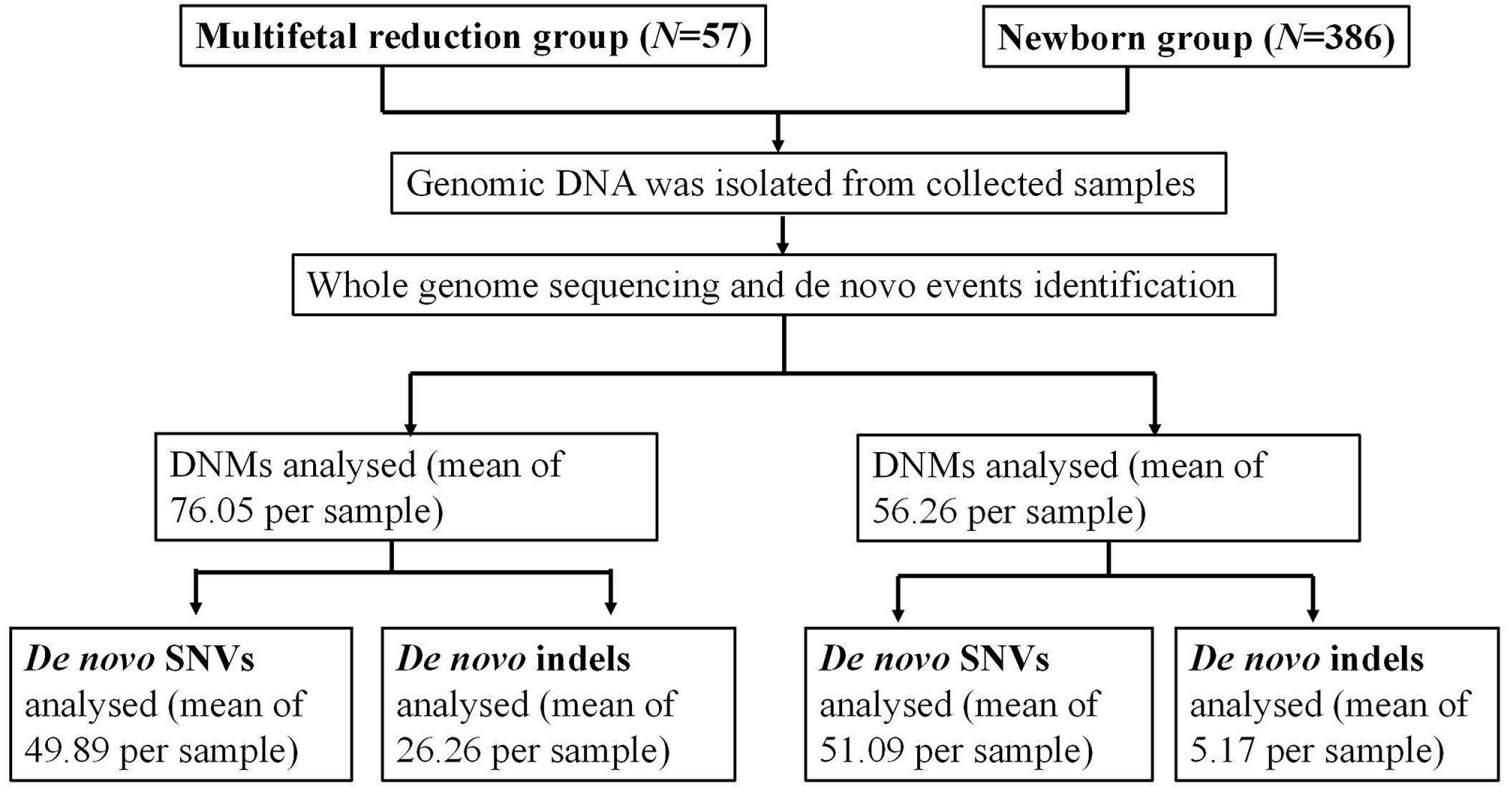
Flowchart of the main results of the study

As illustrated in Fig. 1, Our research encompassed a total of 57 fetuses and 386 neonates, selected in accordance with the corresponding criteria. Based on clinical features, we established two distinct groups: the multifetal reduction group and the newborn group. The clinical background information of both groups is presented in Table 1. The parental age for the newborn group was 32.16 (31.78–32.53) and 33.88 (33.40–34.36), respectively, which was slightly higher than that of the multifetal reduction group. The maternal pre-pregnancy body mass index (BMI) in the multifetal reduction group was 22.14 (21.34–22.94) and in the newborn group was 21.49 (21.22–21.75). There was no significant difference in the age of the parents or the maternal pre-pregnancy BMI between the two groups (*P* > 0.05). The distribution of the DNMs between the two groups is discussed below.

**Table 1:** Parental characteristics between the two groups

	Multifetal reduction group (*N* = 57)	Newborn group (*N* = 386)	F	*P* value
	Mean(95%CI)			
Maternal age (y)	29.84 (29.05–30.64)	32.16 (31.78–32.53)	3.55	0.06
Paternal age (y)	32.25 (31.23–33.26)	33.88 (33.40-34.36)	2.82	0.09
Maternal pre-pregnancy BMI (kg/m^2^)	22.14 (21.34–22.94)	21.49 (21.22–21.75)	3.43	0.07

Note: BMI = body mass index; ICSI = intracytoplasmic sperm injection

### *De novo* events in the progeny from developing fetus to birth subsequent to implantation

The sequencing results show a WGS depth of 30X ∼ 40X per sample, yielding approximately 120G of data and sequencing quality of Q20 > 95% and Q30 > 85%. Based on the quantity of bases affected by the mutation, DNMs can be classified into categories: SNVs and indels. For the purposes of this study, only *de novo* SNVs and indels were analyzed. The genomic region in which DNMs occur can be divided into ten distinct groups based on their biological function including downstream, exonic, intergenic, intronic, ncRNA-exonic, ncRNA-intronic, splicing, upstream, UTR3, and UTR5. In this study, we focused on three major genetic regions, which were analyzed as follows: the downstream and upstream regions were classified as intergenic regions, while the ncRNA-intronic and splicing regions were categorized as intronic regions. Finally, the ncRNA-exonic, UTR3, and UTR5 regions were grouped as exonic regions.

**Fig. 2:**
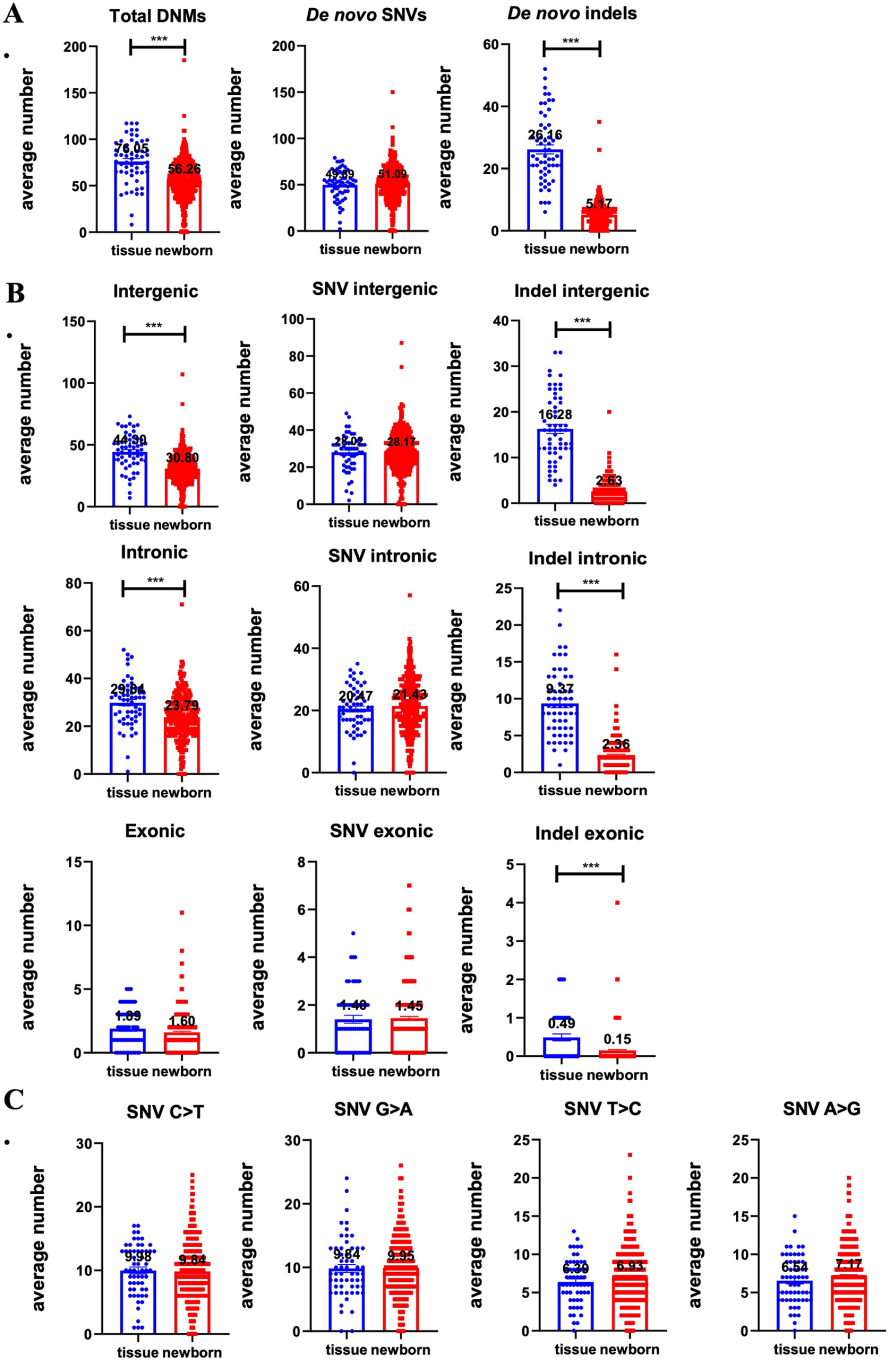
The average number of DNMs catalogued between the multifetal reduction group and the newborns. (**A**) The occurrence of the average number of total DNMs, *de novo* SNVs and *de novo* indels in the two groups. The results revealed a statistically significant decrease in the average number of total DNMs and *de novo* indels in newborns (*P* < 0.001). (**B**). The distribution of various genome regions in total DNMs, *de novo* SNVs and *de novo* indels. The average number of total DNMs were significantly differences between the two groups in both intergenic and intronic regions. There were significantly differences in the number of *de novo* indels across all regions examined (*P* < 0.001). (**C**) The distribution of various mutation modes in *de novo* SNVs, specifically C > T, G > A, A > G, and T > C, was analyzed. No significant differences were noted in the frequency of base exchange between the two groups (*P* > 0.05). *** indicates *P* < 0.001

The average number of total DNMs in the newborns was observed to be 56.26 (54.17–58.35), which appeared to be lower than that in the multifetal reduction group, which was 76.05 (69.70–82.40) (F = 2.42, *P* = 0.12). However, after adjusting for parental age and maternal pre-pregnancy BMI, significant differences were observed between the two groups (F = 50.60, *P* < 0.001). Furthermore, we categorized the total number of DNMs into various genetic regions and found that the distribution of DNMs in genome sections varied. Notably, the number of DNMs per capita in intergenic and intronic regions was found to be the highest among all the groups. The average number of DNMs in intergenic regions was found to be 44.30 (40.47–48.13) in the multifetal reduction group and 30.80 (29.59–32.02) in the newborns (F = 3.72, *P* = 0.05). Similarly, the average number of DNMs in intronic regions was 29.84 (27.23–32.46) in the multifetal reduction group and 23.79 (22.84–24.74) in the newborns (F = 0.05, *P* = 0.83). After adjusting for parental age and maternal pre-pregnancy BMI, it was determined that the observed differences were statistically significant. The average number of DNMs in exonic regions was 1.89 (1.49–2.30) in the multifetal reduction group and 1.60 (1.45–1.75) in the newborns (F = 0.86, *P* = 0.35).There is no difference between groups in statistics. These findings are presented in Fig. 2; Table 2.

**Table 2:** The average number of DNMs catalogued between the multifetal reduction group and the newborns

	Multifetal reduction group (*N* = 57)	Newborn group (*N* = 386)	F	*P*	Adjusted F	Adjusted *P*
	Mean(95%CI)					
DNMs	76.05 (69.70–82.40)	56.26 (54.17–58.35)	2.42	0.12	50.60	0.00
*De novo* SNVs	49.89 (45.59–54.20)	51.09 (49.22–52.96)	1.01	0.32	0.91	0.76
*De novo* indels	26.26 (23.27–29.05)	5.17 (4.82–5.52)	194.17	0.00	807.29	0.00
DNMs in intergenic	44.30 (40.47–48.13)	30.80 (29.59–32.02)	3.72	0.05	68.05	0.00
DNMs in intronic	29.84 (27.23–32.46)	23.79 (22.84–24.74)	0.05	0.83	24.27	0.00
DNMs in exonic	1.89 (1.49–2.30)	1.60 (1.45–1.75)	0.86	0.35	2.04	0.15
*De novo* SNVs in intergenic	28.02 (25.48–30.55)	28.17 (27.09–29.25)	0.97	0.33	0.45	0.50
*De novo* SNVs in intronic	20.47 (18.59–22.36)	21.43 (20.57–22.29)	3.82	0.05	0.02	0.88
*De novo* SNVs in exonic	1.40 (1.07–1.74)	1.45 (1.31–1.59)	0.15	0.70	0.001	0.97
*De novo* indels in intergenic	16.28 (14.31–18.26)	2.63 (2.41–2.85)	234.77	0.00	781.90	0.00
*De novo* indels in intronic	9.37 (8.18–10.55)	2.36 (2.17–2.55)	95.61	0.00	398.57	0.00
*De novo* indels in exonic	0.49 (0.31–0.67)	0.15 (0.11–0.20)	45.12	0.00	21.17	0.00
*De novo* SNVs of C > T	9.98 (8.89–11.07)	9.84 (9.39–10.29)	0.18	0.67	0.51	0.47
*De novo* SNVs of G > A	9.84 (8.58–11.1)	9.95 (9.50-10.41)	0.03	0.87	0.14	0.71
*De novo* SNVs of A > G	6.54 (5.71–7.38)	7.17 (6.81–7.53)	0.71	0.40	1.04	0.31
*De novo* SNVs of T > C	6.39 (5.60–7.17)	6.93 (6.57–7.29)	2.54	0.11	0.18	0.68

### Distribution of *de novo* SNVs

After conducting phased DNMs in two cohorts, we proceeded to perform similar workflows on *de novo* SNVs and indels separately. Our findings revealed that tthe average number of *de novo* SNVs associated with the multifetal reduction group and the newborns was 49.89 (45.59–54.20) and 51.09 (49.22–52.96), respectively. Notably, no significantly differences were observed between the groups (F = 1.01, *P* = 0.32). Furthermore, we subdivided the total number of *de novo* SNVs into different genetic regions and found no significant difference between the groups.

Out of the various modes of mutation, the four primary types, namely, C > T, G > A, A > G, and T > C, demonstrated the highest frequency of occurrence, with frequencies of 9.84 (9.39–10.29), 9.95 (9.50-10.41), 7.17 (6.81–7.53), and 6.93 (6.57–7.29) per newborn, respectively. Similarly, with frequencies of 9.98 (8.89–11.07), 9.84 (8.58–11.1), 6.54 (5.71–7.38) and 6.39 (5.60–7.17) per fetus in the multifetal reduction group, respectively. There was no significant difference observed in the frequency of base exchange between the two groups.

The extant literature reports indicate a correlation between DNMs and paternal age. Our own observations, after adjusting for parental age and maternal pre-pregnancy BMI using a multivariable regression model, reveal a similar directionality between the two groups. These findings are presented in Fig. 2; Table 2.

### Distribution of *de novo* indels

It appears that indels occur at lower frequencies than SNVs, likely due to their larger size which collectively affects more base pairs. The average number of *de novo* indels among the multifetal reduction group and the newborns was 26.26 (23.27–29.05) and 5.17 (4.82–5.52) respectively, and a statistically significant difference was observed (F = 194.17, *P* < 0.001). The study also found significant differences in the number of *de novo* indels in intergenic, intronic, and exonic regions, which remained significant even after correcting for clinical background information. The multifetal reduction group had an average of 16.28 *de novo* indels in intergenic regions, 9.37 in intronic regions, and 0.49 in exonic regions. In contrast, newborns had averages of 2.63, 2.36, and 0.15, respectively. All comparisons were statistically significant (*P* < 0.001). These results are presented in Fig. 2; Table 2.

### Parental characteristics when divided by the number of DNMs within the group

In this study, we categorized the parental age and the maternal pre-pregnancy BMI within the multifetal reduction group or newborn group based on the number of DNMs, with the number of 60 as the cutoff point, to investigate whether there were any differences. Within the group of multifetal reduction, there was no statistically significant difference in parental age and the pre-pregnancy BMI between those with less than 60 DNMs and those with 60 or more DNMs. However, in the newborn group, The maternal age for the group with 60 or more DNMs was 33.14 (29.39–36.89), while the paternal age was 34.99 (30.16–39.82). This represented a significant increase in comparison to those individuals who had fewer than 60 DNMs (F = 4.09, *P* < 0.001; F = 4.67, *P* < 0.001, respectively) (Table 3).

**Table 3:** Comparison of parental characteristics devided by the number of DNMs

	Divided by the number of DNMs		F	*P* value
	< 60	≥ 60		
Multifetal reduction group (*N* = 57)	*N* = 11	*N* = 46		
Maternal age (y)	28.82 (25.60-32.04)	30.09 (27.16–33.02)	0.15	0.88
Paternal age (y)	32.09 (28.39–35.79)	32.28 (28.39–36.17)	1.27	0.21
Maternal pre-pregnancy BMI (kg/m^2^)	21.73 (18.66–24.80)	22.24 (19.20-25.28)	0.49	0.62
Newborn group (*N* = 386)	*N* = 218	*N* = 168		
Maternal age (y)	31.39 (27.82–34.96)	33.14 (29.39–36.89)	4.09	0.00
Paternal age (y)	33.02 (28.45–37.59)	34.99 (30.16–39.82)	4.67	0.00
Maternal pre-pregnancy BMI (kg/m^2^)	21.36 (18.65–24.07)	21.65 (19.18–24.12)	1.09	0.28

### Categorical on the pathogenicity of the DNMs

**Fig. 3:**
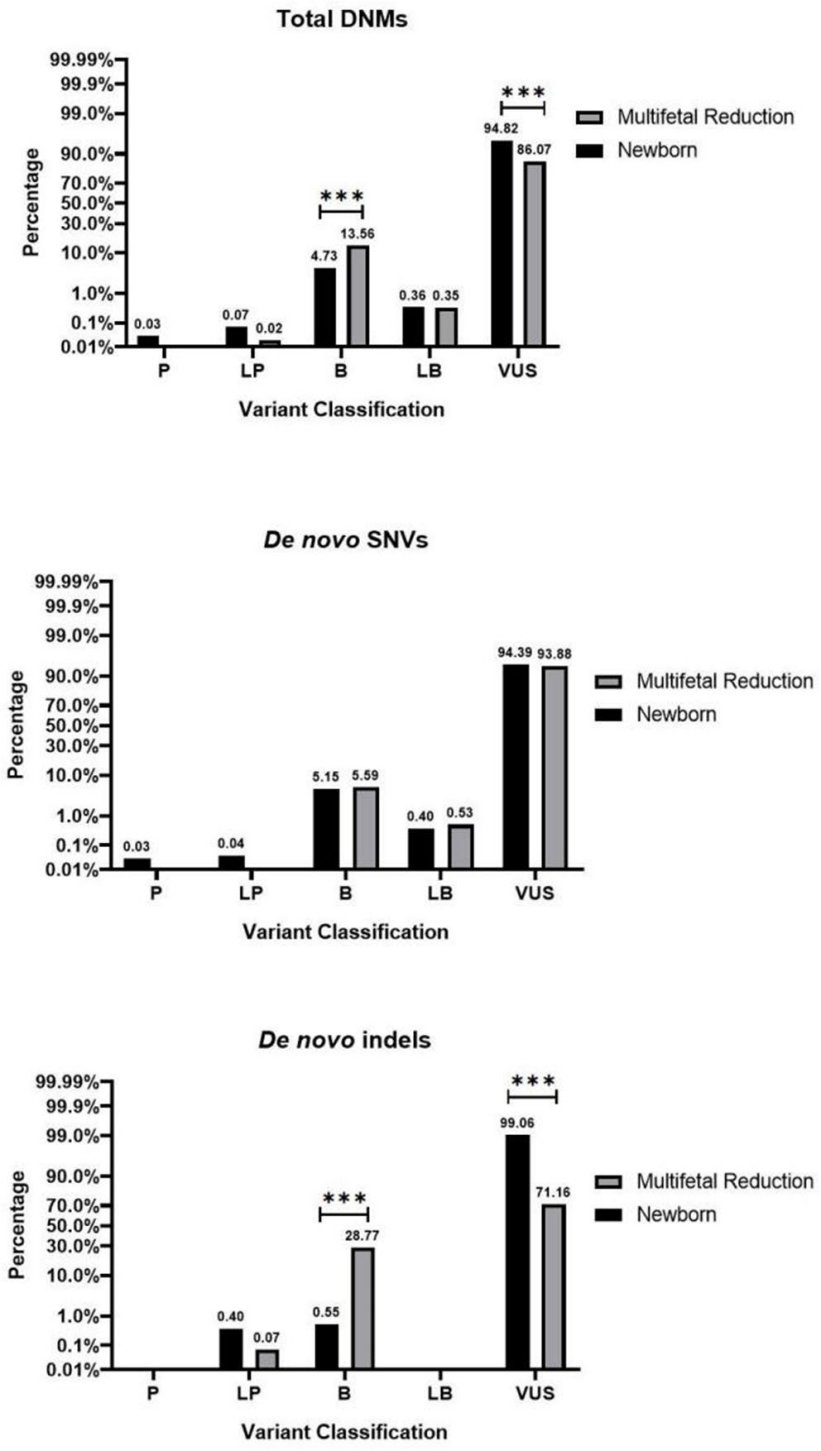
The pathogenicity of DNMs catalogued between the multifetal reduction group and the newborns. Among the total DNMs and *de novo* indels, there were statistically significant differences in both variants of uncertain significance and benign results between the two groups (*P* < 0.001). However, no significant difference was observed in the five pathogenicity classifications of the *de novo* SNVs between the two groups (*P* > 0.05). *** indicates *P* < 0.001. *Note* P = Pathogenic; LP = Likely Pathogenic; B = Benign; LB = Likely Benign; VUS = Variant of Uncertain Significance

Next, we conducted a variant interpretation using InterVar on all of the identified variants. Upon analysis, no significant differences were observed across all groups in terms of pathogenic and likely pathogenic mutations. The pertinent data are presented in Fig. 3, while cases harboring pathogenic or likely pathogenic mutations are enumerated in Table 4.

**Table 4:** Summary of pathogenic or likely pathogenic DNMs detected

Group	Sample	Chr	Position	Ref	Alt	Location	Consequence	Ref.Gene	Interpretation	OMIM
Multifetal reduction group										
	IVF_34	1	229,568,595	G	GCT	Exonic	Frameshift	*ACTA1*	LP	102,610
Newborn group										
	ICSI_11	19	3,983,288	C	CAGTTG	Exonic	Frameshift	*EEF2*	LP	130,610
	ICSI_11	19	6,702,590	C	CTCCTGGCCAGGCCCAGGTGGCTGGCCCGCGCGTG	Exonic	Frameshift	*C3*	LP	120,700
	ICSI_11	22	36,684,985	C	CA	Exonic	Frameshift	*MYH9*	LP	160,775
	ICSI_48_B	8	87,591,395	TC	T	Exonic	Frameshift	*CNGB3*	LP	605,080
	ICSI_20	1	151,396,424	C	A	Splicing	.	*POGZ*	P	614,787
	ICSI_33	22	41,924,576	G	A	Exonic	Nonsynonymous	*ACO2*	LP	100,850
	ICSI_35	1	12,398,359	A	T	Exonic	Stopgain	*VPS13D*	P	608,877
	ICSI_11_B	9	140,851,226	A	T	Exonic	Nonsynonymous	*CACNA1B*	LP	601,012
	ICSI_F_49	21	35,138,202	A	G	Exonic	Nonsynonymous	*ITSN1*	LP	602,442
	ICSI_F_52	2	179,585,709	G	T	Exonic	Stopgain	*TTN*	LP	188,840
	ICSI_F_64	3	119,666,195	G	A	Exonic	Stopgain	*GSK3B*	P	605,004
	IVF_22_B	12	122,701,409	T	TAAAC	Exonic	Frameshift	*DIABLO*	LP	605,219
	IVF_65	13	58,209,060	TC	T	Exonic	Frameshift	*PCDH17*	LP	611,760
	IVF_11	12	104,461,873	ATAATGTTCCC	A	Exonic	Frameshift	*HCFC2*	LP	607,926
	IVF_15	1	162,746,131	C	T	Exonic	Nonsynonymous	*DDR2*	LP	191,311
	IVF_42	15	59,179,576	G	A	Exonic	Stopgain	*SLTM*	P	.
	IVF_43	2	152,421,564	G	T	Exonic	Stopgain	*NEB*	LP	161,650
	IVF_F_81	16	89,351,960	CT	C	Exonic	Frameshift	*ANKRD11*	LP	611,192
	IVF_F_70	4	57,871,884	G	C	Exonic	Nonsynonymous	*POLR2B*	LP	180,661
	IVF_F_96	6	52,101,967	C	G	Splicing	.	*IL17F*	P	606,496
	IVF_F_33	19	36,582,178	C	A	Exonic	Stopgain	*WDR62*	P	613,583

Note: LP = Likely pathogenic, P = Pathogenic

Among the total DNMs, 86.07% and 94.82% of the variants were interpreted as having uncertain significance in the multifetal reduction group and the newborn group, respectively (F = 447.77, *P* < 0.001). 13.56% and 4.73% of the variants were interpreted as benign, respectively (F = 487.05, *P* < 0.001). There were statistically significant differences in both variants of uncertain significance and benign results between the two groups. Among the *de novo* SNVs, there was no difference in the five pathogenicity classifications between the two groups. Among the *de novo* indels, 71.16% and 99.06% of the variants were interpreted as having uncertain significance in the multifetal reduction group and the newborn group respectively, with a significant difference (F = 598.09, *P* < 0.001). Additionally, 28.77% and 0.55% of the variants were interpreted as benign, respectively, also showing a significant difference (F = 622.987, *P* < 0.001).

## Discussion

The occurrence of spontaneous DNMs in the germline is known to promote evolution by providing material for natural selection to act upon. The distribution and frequency of DNMs across the genome contribute to virtually every aspect of an organism’s function and fitness [17]. Spontaneous abortion occurs in 8–20% of recognized pregnancies and typically occurring in the first trimester (7–11 weeks) [18]. Recent research reports suggest that mutations in genes responsible for proper fetal development are a more frequent cause of reproductive failures than chromosomal aberrations. Developmental disorders caused by DNMs have an average prevalence of 1 in 213 to 1 in 448 births, depending on parental age [19]. The study conducted by Kowalczyk et al. has revealed that up to 17.1% of small aberrations cannot be identified through conventional chromosome analysis. These aberrations include genes important for fetal development, and their mutations could cause spontaneous abortion [20]. However, there is a dearth of published research on DNMs that vary from the fetus to the newborn.

Our study was the first to show a significant reduction in the number of *de novo* indels in offspring compared to early pregnancy embryos, across all genomic regions. The differences were statistically significant even after adjusting for factors like parental age and other clinical backgrounds. The estimated indel mutation rate is approximately 4 × 10^–10^ per position, leading to roughly three new indels per genome per generation [21]. Data from Lithuania’s general population, using whole-exome sequencing (WES), showed a *de novo* indel mutation rate of 1.77 × 10 ^− 8^ per position per generation [22]. We found that the frequency of de novel indels was about 26.16 per genome per generation in post-implantation early pregnancy embryos, which was approximately five times higher than that in the newborns (5.17 per genome per generation). It was observed that fetal mutation rates were about fivefold higher than in tissue matched adult stem cells [23]. Elevated mutation accumulation rates are common in fetal cells of various tissues, suggesting that rapid cellular expansion during development is associated with increased mutation accumulation.

Research on *de novo* indels is somewhat limited compared to that on *de novo* SNVs, primarily due to the current WGS technology’s limited accuracy in detecting *de novo* indels [24, 25]. Recent large-scale exome sequencing projects found that *de novo* indels can can lead to many different diseases, ranging from complex neurological diseases to rare Mendelian disorders [26, 27]. The study also shows that *de novo* indels could potentially harm the fetus. The presence of these indels after embryo implantation may be key to fetal health. The obstetrical epidemiological data indicates that the incidence of abortion is approximately 12% [28]. In this study, the total number of *de novo* indels in 9 families during the first trimester exceeded 40, resulting in an incidence of about 16% (9/57). Interestingly, none were found in the newborns with a number exceeded 40, while only two newborns had a total number of *de novo* indels exceeding 20, 26, and 35, respectively. While the embryo’s intrinsic characteristics or the mother’s physiological and biochemical composition may explain this phenomenon, fetuses with a high number of *de novo* indels did not survive later stages of prenatal development. Natural selection mechanism had a boundary in the selection of *de novo* indels during pregnancy. Embryos with a high frequency of *de novo* indels in their genome may be eliminated through fertilization, embryonic development, and selective growth. Another possible reason is that during fetal reduction, a relatively smaller gestational sac may be selected for reduction. Fetuses from smaller gestational sacs may inherently have a higher possibility of mutations and are more likely to be eliminated during later development. However, in our study, there were significant differences in benign variations of *de novo* indel in the fetuses compared to those in the newborns, while the variations in uncertain significance are more significant in the newborns. This phenomenon requires further research to clarify.

Previous research utilized WES to examine deceased fetuses with ultrasound anomalies, revealing diagnostic genetic variants in 20% of cases [29]. Another study evaluated diagnostic or potentially clinically relevant genetic variant in genes associated with developmental disorders in 12.5% of fetuses with structural abnormalities after 11 weeks of gestation [30], many genetic variants in this study were caused by new mutations. Nevertheless, the available literature detailing the pathogenesis that may lead to fetal demise remains insufficient in providing a comprehensive list of mutations or genes responsible for this process. In this study, we selected offspring conceived through ART. There is no evidence of increased DNM load or altered mutation spectrums in mice or humans born through ART compared to spontaneously conceived offspring [31]. Our research team also conducted the same analysis on early artificial abortion tissue and offspring born through natural conception, and found consistent trends (not yet published). However, it is widely acknowledged that infertility itself is linked to unfavorable outcomes in children [32]. To confirm this conclusion, larger sample sizes and core family sequencing results are required, and a thorough characterization of the mechanisms underlying *de novo* indel genesis and evolution in the human genome is necessary.

Our results show no significant difference in the frequency of *de novo* SNVs between the two groups.There are also no significant differences in all genomic regions, including intergenic, intronic, and exonic areas. It is suggested that the occurrence of *de novo* SNVs is mainly determined after successful implantation. Previous studies on humans have shown that approximately 73–78% of *de novo* SNVs are of paternal origin [33]. The investigators noted a rise in the overall count of *de novo* SNVs, specifically 1.28 *de novo* SNVs per year of paternal age and 0.35 *de novo* SNVs per year of maternal age. Additionally, both paternal and maternal age were found to be significantly linked to the number of *de novo* indels, with an increase of 0.071 *de novo* indels per year of paternal age and a smaller increase of 0.019 *de novo* indels per year of maternal age [34]. In this study, the parental age represented a significant increase in comparison to those individuals who had fewer than 60 DNMs in the newborn group.

In the process of interpreting our findings, it is important to acknowledge the limitations of our study. In this study, there was natural heterogeneity in the selection of samples. The embryonic tissue aspirated from the multifetal reduction group were fetal tissues, indicating sample heterogeneity with neonatal cord blood. Report has shown that mutation rates during prenatal development seem to vary among tissues [23]. Another noteworthy aspect is the fact that we were unable to distinguish the infertility factors for the rare samples of multifetal reduction group. Moreover, we could not fully investigate the parental origin of DNMs, only approximately 26% of DNMs in our study could be successfully phased and parent-of-origin called. It has been reported that 15–20% of DNMs can be successfully phased and parent-of-origin called by short-read WGS of parent-offspring trios [35]. Therefore, further exploration is required to improve the detectable rate. One potential approach to achieve this is through the use of multiplexed long-read sequencing [36]. Moreover, it is hard to figure out how those DNMs were associated with the phenotype, and the absence of information about children’s diseases restricted the clinical significance of assessing those DNMs.

## Conclusions

Collectively, we have demonstrated that the occurrence of *de novo* SNVs in offspring is essentially determined after successful implantation. The quantity of *de novo* indels in neonates experiences a significant reduction in comparison to that in early pregnancy embryos post-implantation, and this trend is evident across all genomic regions, underscoring the adverse influence of *de novo* indels on fetal growth after implantation. Nevertheless, to validate these findings further, incorporating a wider range of samples in future studies would aid in identifying more nuanced effects and potentially uncover genetic interactions during intrauterine growth and development.

## Data Availability

No datasets were generated or analysed during the current study.

## Abbreviations

CNVs: Copy number variations
SNVs: Single nucleotide variants
DNMs: *De novo* mutations
ART: Assisted reproductive technology
WGS: Whole genome sequence
IVF: In-vitro fertilization
ICSI: Intracytoplasmic sperm injection
VQSR: Variants quality score recalibration
GATK: Genome Analysis Toolkit
gVCF: Genome variant call format files
GQ: Genotype quality
DP: Read depth
CI: Confidence interval
BMI: Body mass index
WES: Whole-exome sequencing
LP: Likely pathogenic
P: Pathogenic

## References

[1] Lynch M, Ackerman MS, Gout JF, Long H, Sung W, Thomas WK (2016). Genetic drift, selection and the evolution of the mutation rate. Nat Rev Genet.

[2] Ellegren H, Galtier N (2016). Determinants of genetic diversity. Nat Rev Genet.

[3] Noyes MD, Harvey WT, Porubsky D, Sulovari A, Li R, Rose NR (2022). Familial long-read sequencing increases yield of *de novo* mutations. Am J Hum Genet.

[4] Xiang H, Wang C, Pan H, Hu Q, Wang R, Xu Z (2021). Exome-sequencing identifies novel genes Associated with recurrent pregnancy loss in a Chinese cohort. Front Genet.

[5] Jia CW, Wang L, Lan YL, Song R, Zhou LY, Yu L (2015). Aneuploidy in Early Miscarriage and its related factors. Chin Med J (Engl).

[6] Hyde KJ, Schust DJ (2015). Genetic considerations in recurrent pregnancy loss. Cold Spring Harb Perspect Med.

[7] Xie P, Zhang S, Gu Y, Jiang B, Hu L, Tan YQ (2022). Non-invasive preimplantation genetic testing for conventional IVF blastocysts. J Transl Med.

[8] Li X, Hao Y, Chen D, Ji D, Zhu W, Zhu X (2021). Non-invasive preimplantation genetic testing for putative mosaic blastocysts: a pilot study. Hum Reprod.

[9] Popovic M, Dhaenens L, Boel A, Menten B, Heindryckx B (2020). Chromosomal mosaicism in human blastocysts: the ultimate diagnostic dilemma. Hum Reprod Update.

[10] Smits RM, Xavier MJ, Oud MS, Astuti GDN, Meijerink AM, de Vries PF (2022). *De novo* mutations in children born after medical assisted reproduction. Hum Reprod.

[11] Brandes N, Linial N, Linial M (2019). Quantifying gene selection in cancer through protein functional alteration bias. Nucleic Acids Res.

[12] Weber JA, Aldana R, Gallagher BD, Edwards JS (2016). Sentieon DNA pipeline for variant detection - Software-only solution, over 20× faster than GATK 3.3 with identical results. PeerJ PrePrints.

[13] McKenna A, Hanna M, Banks E, Sivachenko A, Cibulskis K, Kernytsky A (2010). The genome analysis Toolkit: a MapReduce framework for analyzing next-generation DNA sequencing data. Genome Res.

[14] Ramu A, Noordam MJ, Schwartz RS, Wuster A, Hurles ME, Cartwright RA (2013). DeNovoGear: *de novo* indel and point mutation discovery and phasing. Nat Methods.

[15] Wei Q, Zhan X, Zhong X, Liu Y, Han Y, Chen W (2015). A bayesian framework for *de novo* mutation calling in parents-offspring trios. Bioinformatics.

[16] Li Q, Wang K, InterVar (2017). Clinical interpretation of genetic variants by the 2015 ACMG-AMP guidelines. Am J Hum Genet.

[17] Yoder AD, Tiley GP (2021). The challenge and promise of estimating the *de novo* mutation rate from whole-genome comparisons among closely related individuals. Mol Ecol.

[18] Bug S, Solfrank B, Schmitz F, Pricelius J, Stecher M, Craig A (2014). Diagnostic utility of novel combined arrays for genome-wide simultaneous detection of aneuploidy and uniparental isodisomy in losses of pregnancy. Mol Cytogenet.

[19] Deciphering Developmental Disorders Study (2017). Prevalence and architecture of *de novo* mutations in developmental disorders. Nature.

[20] Kowalczyk K, Smyk M, Bartnik-Głaska M, Plaskota I, Wiśniowiecka-Kowalnik B, Bernaciak J (2022). Application of array comparative genomic hybridization (aCGH) for identification of chromosomal aberrations in the recurrent pregnancy loss. J Assist Reprod Genet.

[21] Lynch M (2010). Rate, molecular spectrum, and consequences of human mutation. Proc Natl Acad Sci U S A.

[22] Pranckėnienė L, Jakaitienė A, Ambrozaitytė L, Kavaliauskienė I, Kučinskas V (2018). Insights into *de novo* mutation variation in Lithuanian Exome. Front Genet.

[23] Kuijk E, Blokzijl F, Jager M (2019). Early divergence of mutational processes in human fetal tissues. Sci Adv.

[24] Onishi-Seebacher M, Korbel JO (2011). Challenges in studying genomic structural variant formation mechanisms: the short-read dilemma and beyond. BioEssays.

[25] Kloosterman WP, Francioli LC, Hormozdiari F, Marschall T, Hehir-Kwa JY, Abdellaoui A (2015). Characteristics of *de novo* structural changes in the human genome. Genome Res.

[26] Veltman JA, Brunner HG (2012). *De novo* mutations in human genetic disease. Nat Rev Genet.

[27] Takata A (2019). Estimating contribution of rare non-coding variants to neuropsychiatric disorders. Psychiatry Clin Neurosci.

[28] Holt-Kentwell A, Ghosh J, Devall A, Coomarasamy A, Dhillon-Smith RK (2022). Evaluating interventions and adjuncts to optimize pregnancy outcomes in subfertile women: an overview review. Hum Reprod Update.

[29] Yates CL, Monaghan KG, Copenheaver D, Retterer K, Scuffins J, Kucera CR (2017). Whole-exome sequencing on deceased fetuses with ultrasound anomalies: expanding our knowledge of genetic disease during fetal development. Genet Med.

[30] Lord J, McMullan DJ, Eberhardt RY, Rinck G, Hamilton SJ, Quinlan-Jones E (2019). Prenatal Assessment of Genomes and Exomes Consortium. Prenatal exome sequencing analysis in fetal structural anomalies detected by ultrasonography (PAGE): a cohort study. Lancet.

[31] Wood KA, Goriely A (2022). The impact of paternal age on new mutations and disease in the next generation. Fertil Steril.

[32] Bergh C, Wennerholm UB (2020). Long-term health of children conceived after assisted reproductive technology. Ups J Med Sci.

[33] Tatsumoto S, Go Y, Fukuta K, Noguchi H, Hayakawa T, Tomonaga M (2017). Direct estimation of *de novo* mutation rates in a chimpanzee parent-offspring trio by ultra-deep whole genome sequencing. Sci Rep.

[34] Kaplanis J, Ide B, Sanghvi R, Neville M, Danecek P, Coorens T (2022). Genetic and chemotherapeutic influences on germline hypermutation. Nature.

[35] Goldmann JM, Wong WS, Pinelli M, Farrah T, Bodian D, Stittrich AB et al. Parent-of-origin-specific signatures of *de novo* mutations. Nat Genet. 2016;48(8):935-9. Erratum in: Nat Genet. 2018;50(11):1615. 10.1038/ng.3597 27322544

[36] Holt GS, Batty LE, Alobaidi BKS, Smith HE, Oud MS, Ramos L (2022). Phasing of *de novo* mutations using a scaled-up multiple amplicon long-read sequencing approach. Hum Mutat.

